# Omalizumab in chronic spontaneous urticaria (CSU): experience in three cases

**DOI:** 10.1186/1939-4551-8-S1-A155

**Published:** 2015-04-08

**Authors:** Solange Oliveira Rodrigues Valle, Soloni Afra Pires Levy, Sérgio Duarte Dortas, José Elabras Filho, Renata Silva Fernandes, Alfeu Tavares França, Alfeu Tavares França, Cynthia S Monteiro De Castro

**Affiliations:** 1 Hospital Universitário Clementino Fraga Filho Hucff-Ufrj, Brazil; 2 Hospital São Zacharias, Brazil; 3 Universidade Iguaçu, Brazil

## Background

Chronic spontaneous urticaria (CSU) is more common in adults, especially middle-aged women. The condition resolves spontaneously within 6 months in 30 to 55% of patients but can persist for years in others.

It has a devastating effect on the quality of life of those who experience it. Although the mechanisms are not fully elucidated, anti IgE recombinant humanized monoclonal (Omalizumab) has been recommended, according to the latest guidelines EAACI/GA2LEN/EDF & WAO, with encouraging results in management of refractory CSU as opposed to the usual and alternative therapies.

## Methods

We report the use of Omalizumab in three patients presenting CSU attended at Clinical Immunology outpatient service of a Brazilian Reference Center.

## Results

Three women (50, 74 and 28 years old) with a history of urticaria for 10, 7 and 6 years, respectively. Two of them reported intermitent angioedema. Urticaria activity score (UAS) ranged from 4 to 6 in all of them. Laboratory investigation, including autoimmunity and immunodeficiency screening, was normal. Total IgE levels were high in two of them. All three had positive autologous serum skin test (ASST). All of them had used high doses of antihistamines, oral corticosteroids, hydroxychloroquine, dapsone, doxepin, amitriptyline, with no improvement. One of them also used cyclosporine A and intravenous immunoglobulin G for 3 times with a 3 week interval. Infusion was discontinued because patient presented diarrhea, abdominal pain and urticaria exacerbation. Therefore, we decided to use Omalizumab. After treatment consent form signature, they started on 300 mg Omalizumab subcutaneous every 4 weeks.

Patient 1 (50 y) – Showed improvement on the first 48 hours after Omalizumab infusion and has kept UAS = zero until now (for 18 months), without any other therapy

Patient 2 (74 y) – Showed UAS improvement from 6 to 2 after six months using Omalizumab. However, she still needs to use low doses of antihistamines and doxepin.

Patient 3 (28 y) – Showed improvement in the first week treatment, and has kept UAS = zero. She has kept using Omalizumab for 5 months now, but she still needs low doses of antihistamines.

## Conclusions

Our experience has shown Omalizumab to be an effective treatment option for patients with refractory CSU, as directed by the EAACI/GA2LEN/EDF & WAO guidelines.

